# Non-targeted effects of radiation therapy for glioblastoma

**DOI:** 10.1016/j.heliyon.2024.e30813

**Published:** 2024-05-09

**Authors:** Lucie Lerouge, Aurélie Ruch, Julien Pierson, Noémie Thomas, Muriel Barberi-Heyob

**Affiliations:** Department of Biology, Signals and Systems in Cancer and Neuroscience, CRAN, UMR7039, Université de Lorraine, CNRS, 54500 Vandœuvre-lès-Nancy, France

**Keywords:** Glioblastoma, Radiotherapy, Macrophages, Microglia, Abscopal effect, Bystander effect

## Abstract

Radiotherapy is recommended for the treatment of brain tumors such as glioblastoma (GBM) and brain metastases. Various curative and palliative scenarios suggest improved local-regional control. Although the underlying mechanisms are not yet clear, additional therapeutic effects have been described, including proximity and abscopal reactions at the treatment site. Clinical and preclinical data suggest that the immune system plays an essential role in regulating the non-targeted effects of radiotherapy for GBM. This article reviews current biological mechanisms for regulating the non-targeted effects caused by external and internal radiotherapy, and how they might be applied in a clinical context. Optimization of therapeutic regimens requires assessment of the complexity of the host immune system on the activity of immunosuppressive or immunostimulatory cells, such as glioma-associated macrophages and microglia. This article also discusses recent preclinical models adapted to post-radiotherapy responses.

This narrative review explores and discusses the current status of immune responses both locally *via* the "bystander effect" and remotely *via* the "abscopal effect". Preclinical and clinical observations demonstrate that unirradiated cells, near or far from the irradiation site, can control the tumor response. Nevertheless, previous studies do not address the problem in its global context, and present gaps regarding the link between the role of the immune system in the control of non-targeted effects for different types of radiotherapy and different fractionation schemes applied to GBM. This narrative synthesis of the scientific literature should help to update and critique available preclinical and medical knowledge. Indirectly, it will help formulate new research projects based on the synthesis and interpretation of results from a non-systematic selection of published studies.

## Highlights

• This review focuses on the role of the immune system in controlling non-target effects for different types of radiotherapy and according to different fractionation schemes applied to glioblastoma.
• Preclinical and clinical observations demonstrate that unirradiated cells, near or far from the irradiation site, can control the tumor response.
• This review explores and discusses the current status of immune responses both locally via the "bystander effect" and remotely via the "abscopal effect
• The immune environment of glioblastoma, which is highly immunosuppressive, is also described, including glioma-associated macrophages and microglia
• Real-time assessment of cytokine and chemokine secretion before and after treatment may be necessary to monitor the time-dependent immunomodulatory effects on the tumor microenvironment

## Graphical abstract

**Figure:**
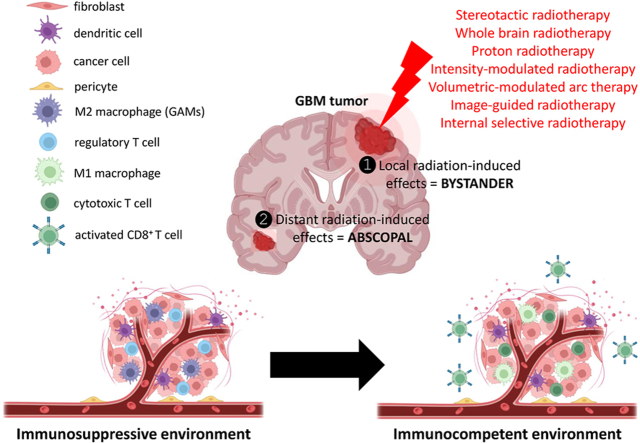

## 1 Introduction

For brain tumors, radiotherapy is considered as a curative treatment for patients with localized cancer and is also used as a palliative strategy for patients with metastatic disease. GBM is a highly aggressive brain tumor which diffusely infiltrates the brain parenchyma and has an extremely poor prognosis. GBM are IDH (isocitrate dehydrogenase)-wild type gliomas according to the 2021 WHO classification of central nervous system (CNS) tumors [1]. It remains particularly difficult to control locally due to the intrusive infiltration of isolated cells into adjoining tissues. This invasive infiltrative disease component is the ultimate cause of recurrence [2]. Standard treatment for patients with newly diagnosed GBM consists of maximal surgical resection followed by postoperative irradiation with concomitant and adjuvant temozolomide therapy [3], however recurrence is almost inevitable [4,5]. At the time of recurrence, treatment options are very limited with modest activity [6]. There is no accepted standard of care for recurrent GBM. The majority of recurrences, according to the *Stupp* protocol, occurs within a 2-cm radius of the previous treatment site [7]. As a result, it is highly likely that any significant improvement in the survival of GBM patients will depend on immune system support to eliminate resistant/residual tumor cells outside the treatment target.

In clinical practice, radiotherapy efficiency is traditionally attributed to the local effects of ionizing radiation, which induce cell death through direct and indirect DNA damage [8], but important research has highlighted an unexpected dual relationship between tumor irradiation and host immune system involvement. Indeed, it is widely accepted that post-radiotherapy effects are mediated by direct damage to DNA, and/or indirect damage due to free radicals generated by water radiolysis. However, this concept has been challenged by numerous observations, demonstrating that non-irradiated cells, whether near or far from the irradiation site, can sometimes undergo the same responses as those originating from the tumor tissue; it has been established that cancer cells exposed to ionizing radiation can release mediators that may influence non-irradiated cells behavior [9].

A distinction can be established between radiation-induced abscopal effect, which is a distal systemic effect (several tens of centimeters outside the irradiated field), mediated by immunogenic responses, and bystander effect, which is characterized by a local communication effect over few millimeters at the treated site, mediated by soluble factors secretion or *via* expression of gap junction proteins, as well as inflammatory cells activation from tumor microenvironment [10,11]. However, our understanding of the impact of radiation on immune system activation is still in its infancy, and challenges for therapeutic applications have yet to be overcome.

This review explores the current status of the immune responses both locally through bystander effect and, distantly, through abscopal effect. To understand key factors involved in these effects, immune environment of GBM, which is recognized as highly immunosuppressive, is described. Limited information is available on the activity of immunosuppressive or immunostimulatory cells, including glioma-associated macrophages and microglia (GAMs). In fact, several studies have demonstrated that microglia and macrophages account for almost half of cells constituting GBM tumor mass [12,13] and, preclinical models adapted to post-radiotherapy responses are being studied to identify the source of inflammatory mediators that may alter cell dynamics and molecular pathways involved in tumor recurrence. Stereotactic microdialysis is suggested as a method for real-time assessment of various cytokines and chemokines involved in GBM immunologic processes. These findings may lead to identify new molecular targets and inflammatory mediators. Additionally, it is crucial to clarify whether the immune response is localized in the tumor tissue or in the brain adjacent to tumor.

## 2 Understanding GBM immune microenvironment

**Fig. 1:**
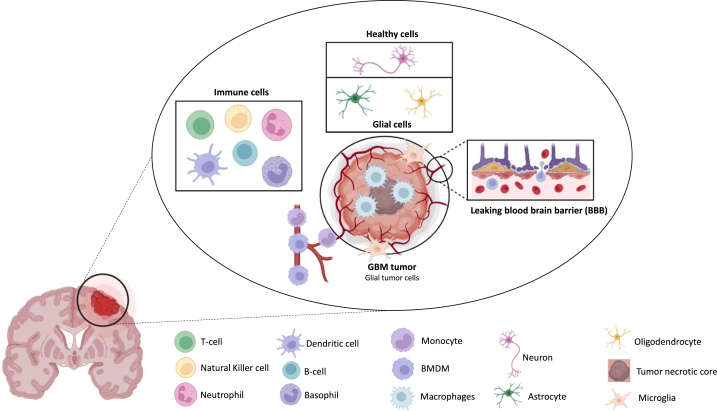
GBM microenvironment. Various cell types (tumor, immune, and glial cells), brain vasculature and extracellular matrix. Macrophages originate from several sources: microglia already present into tumor tissue, monocytes recruited *via* blood vessels and BMDM (also *via* vasculature). Macrophages are located close to the necrotic area. Astrocytes, along with endothelial cells and pericytes, contribute to maintain blood-brain barrier, but in GBM this barrier loses its integrity, leading to leakage (adapted from [[106], [107]]).

GBM microenvironment is characterized by a variety of cell types, most notably astrocytes, oligodendrocytes and neurons, but also immune cells and brain vasculature. The immune defense of the CNS is determined by brain-resident macrophages and microglia, both of which are effectors of the innate immune response (Fig. 1). GAMs are the predominant immune population in GBM, representing up to 30–50 % of tumor cells [14]. Based on their molecular signatures, parenchymal microglial cells and recruited monocyte-derived macrophages exhibit disease-specific phenotypic characteristics. Macrophages are localized in perivascular and necrotic regions whereas microglia is more abundant in peritumor regions [15].

**Fig. 2:**
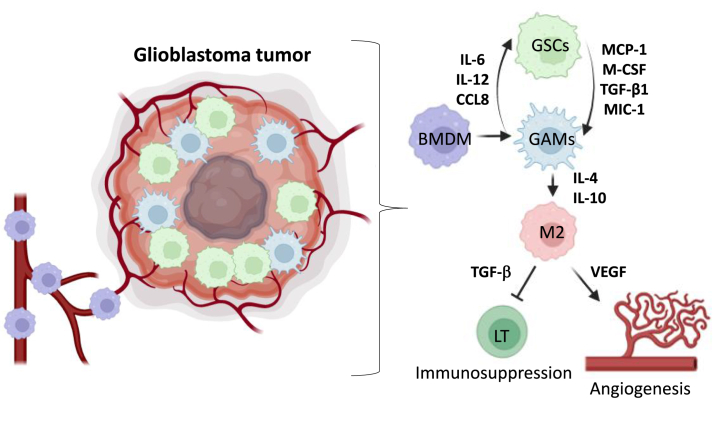
GAMs impact on immune modulation in GBM. BMDM are recruited by GSCs by chemoattraction *via* MCP-1 and M-CSF, controlling macrophages differentiation. Macrophages can also have an impact on GSCs, by secreting stemness factors. Within GBM microenvironment, GAMs are polarized to a M2 phenotype through secretion of cytokines. This polarization leads to immune suppression and angiogenesis *via* secretion of TGF-β and VEGF.

GAMs are recruited to the tumor tissue by a variety of secreted factors, resulting in polarization towards a pro-tumorigenic M2 macrophage phenotype. M2 phenotype can be sustained by autocrine IL-10 signaling. Additionally, GAMs possess an M2-associated secretome which improves extracellular matrix degradation through expression of several matrix metalloproteinases (MMPs), and they also promote angiogenesis. By secreting transforming growth factor (TGF-β), interleukin-6 (IL-6), IL-1β, EGF and IL-10, GAMs facilitate glioma cell growth, invasion, and migration as illustrated in Fig. 2. Macrophages are recruited by chemoattraction mediated by chemokines, such as monocyte chemoattractant protein-1 (MCP-1). The relationship between GMB stem cells (GSCs) density and macrophages suggests that GSCs may recruit macrophages through chemokines (CCL), such as CCL2 (also called MCP-1), CCL5 and CCL7. Macrophages infiltration can lead to TGF-β secretion, promoting angiogenesis and immunosuppression and *in fine* local relapses [16](Fig. 2)**.** It was also shown that GSCs can produce TGF-β1, macrophage inhibitory cytokine-1 (MIC-1) and M-CSF which induce monocytes to differentiate into macrophages [17]**.** On the other hand, macrophages can also regulate GSCs self-renewal by secreting factors supporting stemness such as IL-6 [18], IL-12 [19] and CCL8 [20].

### 2.1 Subtypes of microglia and macrophages in GBM

Many pathological events in CNS can induce polarization of microglia/macrophages. Furthermore, it has been suggested that the immunostimulatory effects of radiotherapy are mediated by modulation of polarized microglia/macrophages; M2 type increases tumor growth and suppresses immune responses, whereas M1 type reduces tumor growth [[21], [22], [23]]. Several studies have demonstrated the benefits of switching from M2 to M1 type to improve the efficacy of radiotherapy [24]. M1 phenotype expresses co-stimulatory molecules, including IL-6, tumor necrosis factor-alpha (TNF-α), chemokine 10 (CXCL10), and major histocompatibility complex (MHC) class II, providing an antigen presentation function [25]. Finally, M1 microglia/macrophages exhibit a classic activated phenotype with well-defined functions, whereas M2 type polarization exhibits a dynamic state [26]. Type-II inflammatory factors such as IL-4, IL-10, and IL-13 induce M2 phenotype polarization, preventing secretion of many anti-inflammatory factors, leading to downregulation of the inflammatory response. Consequently, M2-type microglia/macrophages favor tumor progression [27].

**Fig. 3:**
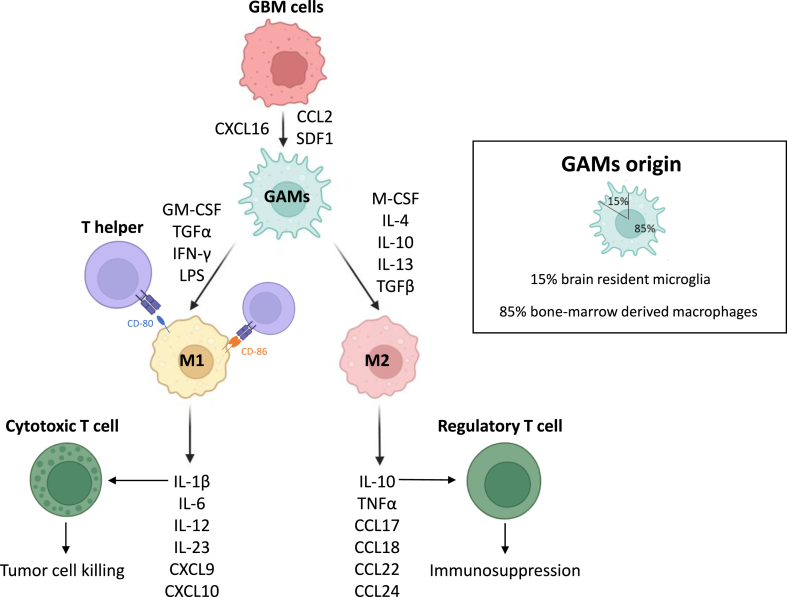
–Interaction between GAMs and glioma cells. GAMs arise from brain resident microglia (15 %) and from BMDM (85 %). Glioma cells can recruit macrophages. Secretion of CXCL16, CCL2, SDF-1 leads to an anti-inflammatory microenvironment. M1 and M2 macrophages have different functions in tumor microenvironment, M1 promoting cytotoxic T-cells trough cytokines secretion such as IL-1β whereas M2 promoting regulatory T-cells by IL-10 secretion. M1 express CD80 and CD86, from the immunoglobulin superfamily, which act as antigens for T-helper cells, leading to immune-mediated cell death.

In the case of GBM, single-cell sequencing studies have shown that some cells may contain genes that promote inflammation (M1 phenotype), while others express genes that promote immunosuppression (M2 phenotype) [28]. These findings challenge the traditional M1/M2 phenotypes and suggest that polarization may be a continuous process; demonstrating the high adaptability of GBM [29,30](Fig. 3).

### 2.2 Impact of blood-brain barrier disruption

After GBM tumor development, BMDM infiltrate brain parenchyma as a result of alterations of blood-brain barrier (BBB) and chemokines release. BBB is significantly altered in GBM, leading to infiltration of adaptive and innate immune cells into the tumor microenvironment, mostly suppressive macrophages and T regulatory lymphocytes (Tregs) [31]. Despite microglia and BMDM have distinct origins, they exhibit similar immune functions and express common markers, including CD68 [32]. Transmembrane protein 119 (TMEM119) was recently discovered as a specific marker for microglia, enabling microglia and macrophage differentiation in both human and mouse models [33]. Furthermore, changes in BBB result in increased expression of suppressive molecules such as programmed cell death ligand 1 (PD-L1). PD-L1 inhibits recruitment of activated T cells [34].

## 3 Immune modulation post-radiation therapies

### 3.1 Radiation therapies applied to GBM

External radiation therapy is a vital treatment modality in GBM management and was widely demonstrated to improve overall survival in numerous clinical trials [35]. A conventional dose of radiotherapy (RT) was established, corresponding to a treatment once a day over 5 days with doses between 1.8 and 2 Gy, resulting in an overall dose of 60 Gy. Three-dimensional conformal radiation therapy (3D-CRT) has gradually replaced "conventional" RT, allowing for better tumor delineation [36]. Previous studies employed basic 2- and 3-D irradiation methods, exposing large areas of healthy brain tissue from moderate to high irradiation levels such as whole brain RT (WBRT), increasing risk for acute and delayed neurological damage [37]. More recently, advanced and customized planning techniques were suggested and implemented, including intensity-modulated radiation therapy (IMRT), image-guided radiation therapy (IGRT), volumetric-modulated arc therapy (VMAT) and stereotactic radiation therapy (SRT). IMRT offers additional ability of adjusting beam intensity to deliver higher doses in a shorter time [38] and IGRT to perform a precise gradient of radiation between tumor volume and adjacent healthy tissue, thanks to medical imaging techniques, allowing a precise delineation. For instance, magnetic resonance imaging (MRI) has become essential in this context [39], whereas functional imaging, using variety positron emission tomography (PET) tracers, is still under consideration [40]. VMAT is an evolution of IMRT, allowing full 360° rotation to achieve continuous radiation beams, which reduces treatment time [36]. SRT involves the use of multiple radiation beams, minimizing neurocognitive deficits risk as the targeted volume is optimal (with a 2 mm margin). This is achieved by Gamma Knife technology, which focuses radiation beams (gamma rays) on the tumor zone [41,42]. For patients with high-grade gliomas, Gamma Knife radiosurgery has demonstrated a significant median overall survival of 12.8 months [43] and 11 months [44]. Finally, applied to recurrent high-grade gliomas, one clinical study reported a median overall survival of 13 months (patients had already benefited from conventional treatment) [45].

Proton RT appears to be as an increasingly accessible approach to limiting off-target irradiation, due to the inherent characteristics of heavy particle irradiation. Additionally, proton RT might offer the most practical means for delivering high-dose FLASH-RT (40 Gy s^−1^ *versus* 0.5–5 Gy min^−1^ for conventional RT) [46]. Moreover, recent advancements in nuclear medicine have also been suggested [47]. Nuclear medicine practitioners classically use β-particle emitters and in the field of GBM, these radionuclide can be linked to nanoparticles, monoclonal antibodies and/or peptides to induce a targeted approach, which is called selective internal radiation therapy. In this context, β-particle emitters (*e.g.* ^131^I, ^90^Y or ^177^Lu) have a wider irradiation range from few millimeters to few centimeters. These physical properties can offer advantages such as high irradiation of tumor margins and "crossfire" effects. The proof-of-concept for GBM targeted internal selective RT using β-emitting isotopes was successfully demonstrated [48]. It is also possible to use α-particle emitters such as 213Bi and 225AC; this is known as targeted alpha therapy (TAT). One of the advantages of alpha emitters over beta emitters is their short range (100 μm vs. millimeters for beta emitters) with higher linear energy transfer (100 keV/μm); reducing damage to healthy cells by delivering an extreme dose only to targeted cells. In addition, alpha emitters can induce DNA double-strand breaks in tumor cells, whereas beta emitters cannot, acting through indirect effects [49]. ^213^Bi (mixed alpha/beta emitter) and ^225^AC (pure alpha emitter) are trivalent metal ions that enable stable binding to biomolecules such as antibodies. Several studies have demonstrated feasibility and low toxicity [50,51]. Clinical studies have been carried out to assess the efficacy of treatment in recurrent GBM [52,53]. The median overall survival was 23.6 months in one study and 16.4 months in the other (*versus* 14.6 months with the Stupp protocol). These studies therefore highlighted improvement of TAT compared to standard therapy. In an additional study, TAT was reported to remodel tumor microenvironment, thus improving immunotherapy efficacy [54]. A decrease in CD4^+^ regulatory T cells and an increase in IL-2, CCL-5 and IFN-γ production, as well as CD8^+^ T cell infiltration, proved anti-tumor effect. Nevertheless, tumor cells increased PD-L1 expression and decreased anti-tumor cytokine production, suggesting that TAT would be effective in combination with anti-PD-L1 immunotherapy which is allowed by the possible stable linking of biomolecules such as antibodies on ^213^Bi (mixed alpha/beta emitter) and ^225^AC (pure alpha emitter) through their trivalent metal ions forms.

### 3.2 Immune modulation as a function of irradiation type and scheme

**Fig. 4:**
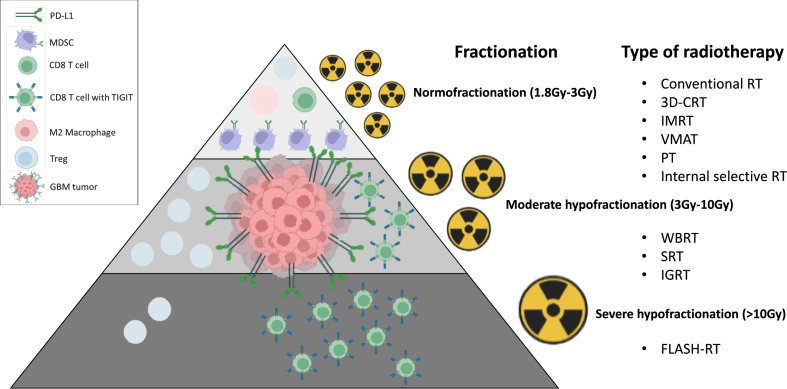
–Immune cells recruitment for different RT fractionation schedules, adapted from (70). In a pre-clinical study comparing three fractionated radiation protocols [108], different immune responses were observed. Each fractionation scheme elicited different lymphoid and myeloid responses. The longest delay in tumor growth was observed with 18 × 2 Gy and 3 × 8 Gy fractionation schemes, compared to 1 × 16.4 Gy. The radiation doses of 3 × 8 Gy and 1 × 16.4 Gy resulted in a lymphoid response, activating CD8^+^ T-cells and regulatory T-cells. In contrast, dose of 18 × 2 Gy induced a myeloid response, activating myeloid-derived suppressor cells and tumor-associated M2 macrophages. CD8^+^ T-cells expressed an increased level of TIGIT when exposed to a dose of 3 × 8 Gy, while a dose of 18 × 2 Gy resulted in a decrease of TIGIT expression. The same results mentioned above were also observed using RNAseq technology. Radiotherapy was significantly more effective when administered at a dose of 3 × 8 Gy compared to other doses. We have associated each different radiation therapy type to a fractionation scheme.

Ability of RT to induce tumor immunogenicity depends not only on the delivered total dose, irradiation type but above all, on the irradiation scheme (Fig. 4).

A study conducted *in vitro* showed that daily doses of 2 Gy irradiation had an impact on immune system characterized by an inflammatory response of myeloid cells after irradiation [55]. *In vivo*, a significant increase in the levels of IL-8, MCP-1, and MIP-1α was observed in the tumor tissue environment post-irradiation. The different proteins MIP-1, MIP-1α (CCL3) and MIP-1β (CCL4) are crucial chemokines for inducing immune responses involved in the inflammatory process. There is also evidence that five 2 Gy irradiation fractions induce an increase in IL-6 expression [55].

Increased levels of IL-6 and IL-8 were also confirmed by another study, highlighting an upregulation of both cytokines at the gene and protein expression 35 days post-using 8 and 12 Gy [56]. The authors demonstrated that approximately one week after treatment (8 Gy RT), an initial proliferative index was obtained with a population doubling time similar to the pre-irradiated population. Conversely, proliferative capacity of samples irradiated at 16 Gy decreased up to three weeks post-irradiation. These results were correlated with inflammatory cytokines and chemokines (IL-6, IL-8, CXCL1 and CXCL5) gene expression. Chemokines CXCL1 and CXCL5 may promote angiogenesis and cancer cells proliferation, migration and invasion. In addition, IL-6 confers radio-resistance and IL-8 facilitates angiogenesis [57].

In GBM, RT using normofractionated doses, has also been described as decreasing recruitment of macrophage subpopulations by assessing CD68 protein expression while increasing MRP-14, a protein 14 related to migration inhibitory factor [58]. Normofractionated RT can also induce phenotypic changes in recruited macrophages, notably a M2 enrichment [59]. Overall, results suggest that X-ray irradiation with low doses induces mitotic catastrophe rather than apoptosis, which is the hallmark of M0 and M1 macrophages recruitment [59]. Moreover, M1 phenotype appears to be more radiosensitive, although results appears controversial [[59], [60], [61]]. Some teams reported an increase in M1 markers after normofractionated doses of RT, while others in M2 markers. These findings could help elucidate GBM recurrence, as M2 macrophages are known to associate with GSCs and, consequently, promote tumor development. These results lead researchers to assert that the increased proportion of M2 in GBM after RT is not due to a change in macrophage phenotype, but rather to the selective disappearance of M0 and M1 [59].

In another study, it was demonstrated that DNA exonuclease Trex1 regulates RT-induced tumor immunogenicity [62] and, irradiation doses higher than 10 Gy resulted in Trex1 induction, reducing cancer cells immunogenicity by degrading accumulated cytosolic DNA. At doses lower than 10Gy, Trex1 is not induced, IFN-β expression being enhanced.

Regarding internal selective RT, absorbed dose is approximatively 100 to 1000 lower than external RT however with a significantly longer exposure time due to α and β radioisotopes [63]. Pre-clinical and clinical findings with low irradiation doses indicated activation of the immune system *via* the cGAS-STING pathway [64]. This pathway results in type I interferon (as IFN-β) expression, activating T cells and leading to extracellular vesicles release. These vesicles activate antigen-presenting cells such as macrophages through to the major histocompatibility complex II (MHCII) receptor [65]. M1 macrophages, in particular, upregulate MHCII [66].

## 4 Non-targeted effects of RT

### 4.1 Bystander effect

**Fig. 5:**
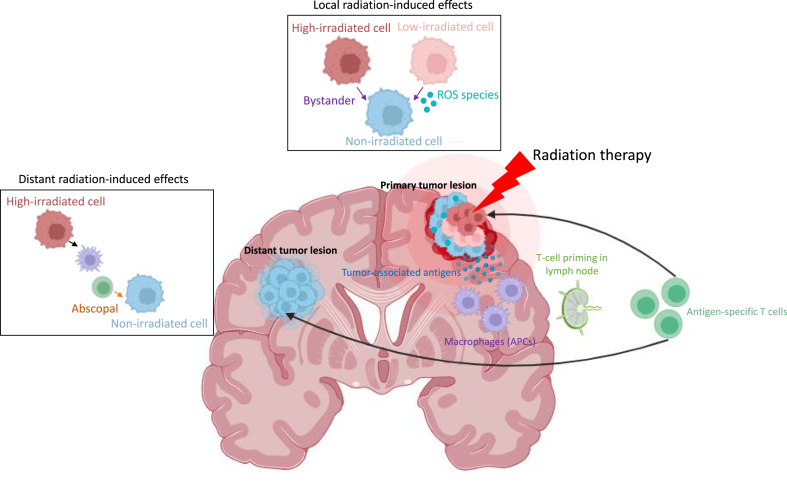
Non-targeted effects post-irradiation for GBM. Indirect effects such as the bystander effect, occurs between high-/low-irradiated cells that have been targeted by irradiation and non-irradiated cells. Activated cells may produce cytokines and/or ROS. As a result, tumor-associated antigens are released, activating immune system, particularly APCs such as macrophages. APCs cross-present tumor antigens to T-cells in lymph nodes. T-cells which recognize specific antigen, attack tumor tissue both within and outside irradiated field.

Direct irradiation induces biological changes in tumor tissue, however neighboring cells may also be affected by a biological response known as the irradiation-induced bystander effect [67]. Short-range proximity effects occur through transmission of intracellular information between cells *via* gap junctions, leading to increased levels of DNA double-strand breaks and cell death [68]. Long-distance indirect effects can be mediated by cytokines secretion, which circulate into the lymphatic drainage and vascular system. Mediators reach regions or tissues distant from the irradiated tumor site [69](Fig. 5).

### 4.2 Abscopal effects

These are anti-tumor consequences, occurring at a distant site from the irradiated one. The term “ab” means “position away” and “scopus” means “target”. Abscopal effects are considered the 6th R of Radiobiology, being the reactivation of anti-tumor immune response [70]. The specific mechanism of abscopal effects requires the relative contribution of APCs such as macrophages or dendritic cells (Fig. 5). Abscopal effects primarily rely on irradiation pattern, radiation dose, and immune control to occur and manifest [71]. Tumor cells death due to irradiation leads to the release of neo-antigens during the abscopal response and before T-cell activation. High irradiation dose stimulates the release of tumor cell fragments, containing immunogenic molecules. When activated, tumor-specific T cells enter the circulation and selectively target tumor cells, promoting regression of unirradiated tumors. The majority of GBM tumors lack neoepitopes, highlighting the importance of developing strategies capable of enhancing the immune response [72]. However, abscopal effects are rarely observed with RT alone [73], and immunotherapies are often associated with reversal of radioresistance related to tumor immunity in "cold" tumors. These tumors are not infiltrated by T cells, and the main tumor immune population consists of pro-tumor microglia/M2 macrophages [74]. A complete understanding of the mechanisms that might amplify the macrophage-dependent abscopal effect is currently required. The release of inflammatory cytokines or molecular patterns associated with tumor cell death has been suggested [75].

### 4.3 Immunogenic cell death post-irradiation

**Fig. 6:**
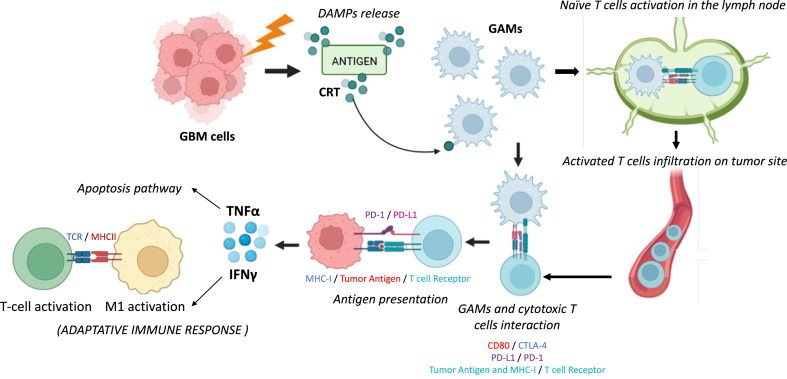
Immunogenic cell death by RT (adapted from (78)). RT can, *via* cell death pathways, reticulum endoplasmic stress response, and autophagy, induce enhanced antigen presentation, proinflammatory cytokine production and cell surface translocation with the release of DAMPs, leading to maturation of APC such as M1 macrophage. They can activate effector immune cells such as CTL *via* interaction of the MHCII with TCR. APC can also travel to regional lymph nodes, priming and activating naive T cells *via* interaction of TCR on T cells and MHC-I, leading to activated T cells infiltration on tumor site.

RT can induce cell death pathways such as apoptosis and mitotic catastrophe [76]. Immunogenic cell death is considered as additional [77], characterized by a pre-apoptotic surface presentation or release of danger-associated molecular patterns (DAMPs). The exposed DAMPs interact with APC, leading to cytotoxic T lymphocytes activation for an adaptive immune response. Therefore, RT-induced immunogenic cell death create a favorable immune environment within tumors to activate immune effector cells [78](Fig. 6).

It has been established that a single dose of 15 Gy, known as ablative dose, can activate naive CD8^+^ T cells *via* antigen presentation. Moreover, high-dose fraction treatments of 6 Gy x 5 and/or 8 Gy x 3 fractions were demonstrated to induce immune-mediated abscopal responses when used in conjunction with anti-CTLA-4 immunotherapy. In addition, treatment with 8 Gy x 3 fractions with CTLA-4 blockade increases tumor-infiltrating CD4^+^ and CD8^+^ lymphocytes [79,80]. However, the effect is significantly weaker for fractions of 3 Gy, and no abscopal effect was demonstrated after hyperfractionated RT (dose below 1.8 Gy).

T-cell activation is not sufficient to eradicate the tumor, but RT can promote lymphocyte infiltration [81] (Fig. 6). To achieve more effective immune modulation, it is crucial to spare circulating regional lymphocytes, improve antigen presentation and activate effector T cells. Studies have shown that X-rays mainly affect M1 macrophages, suggesting that sparing this phenotype can be highly beneficial. Treatment of GBM is also challenging due to tumor hypoxia, and it may be beneficial to target hypoxic parts to preserve immune microenvironment. A single high-dose irradiation aimed at hypoxic regions resulted in abscopal effects [82]. Gamma-ray radiation therapy (with ^137^Cs) induces expression of immune modulators such as the proinflammatory cytokines IL-1β and TNF-α, in addition to IFN-γ. It also enhances the expression of CXCL9, CXCL10 and CXCL16, which attract macrophages [83,84].

For the abscopal effect to be effective, two factors need to be taken into account: firstly, need to activate CD8^+^ T cells, and secondly, sufficient APC to activate CD8^+^ T cells and produce specific killer T cells [85]. To promote abscopal effects, RT could be combined with immunotherapy such as CTLA-4 blockade [86,87]. CTLA-4 binds to APC receptors CD80/CD86, reduces T cell activation and proliferation at GBM tumor site. Several other immune checkpoint inhibitors targeting PD-1/PD-L1, TIM3/GAL9 and TIGIT/CD96 have to be considered [88]. With regard to macrophages, a recent study evaluated their role in radiation-induced abscopal anti-tumor effects [89]. Authors demonstrated that HMGBI, a DAMPs endogenous TLR activator, released from irradiated cancer cells, could promote abscopal M1-macrophages by secreting TNF-α. Interestingly, experiments were performed using SCID mice, lacking adaptative immune response, whereas an abscopal effects were observed. Using SCID mice, an increase in M1 macrophages population was demonstrated in both irradiated and non-irradiated tumors from 14 to 73 % and a decrease in M2 macrophages from 70 to 13 % (see Table 1).

## 5 Clinical trials on radio-immunotherapy for GBM

Ongoing clinical trials combine immune checkpoint inhibitors with hypofractionated RT (Table 2). Clinical trials NCT03532295 and NCT04047706 target IDO1 to avoid immunosuppressive properties (limiting T cell function), while others target the PD-1 receptor to block its interaction with PD-L1. Clinical trial NCT04922723 targets CD-38, which, in hypoxic conditions, can establish an immunosuppressive environment. CD-38 is a multifunctional ecto-enzyme that metabolizes nicotinamide dinucleotide NAD+ and is involved in the homeostasis and extracellular nucleotides, as well as intracellular calcium. CD-38 is also an emerging therapeutic target in certain conditions, preventing the activation of T cells and macrophages. Some of these clinical trials have already been completed (Table 2), and none has achieved a better median progression-free survival or a better median overall survival compared to the classic Stupp protocol.

**Table 1:** Relationships between types of RT, fractionation schemes, total doses delivered, radiation quality and occurrence of bystander/abscopal effects.

Types of RT	Fractionation scheme	Total dose(Gy)	Radiation quality	Bystander/abscopal effect(s)
Proton therapy	1,7 Gy (56 fractions)	96,6	Protons	Pro-inflammatory factors secretion (such as IL6, IL-8, MCP-1 and MIP-1α), which favor migration and maturation of immune cells, activate cGAS-STING pathway and increase anti-tumor response.
Conventional RT	1,8-2 Gy (5 fractions/week over 6 weeks)	60	X-rays	
3D-CRT	2 Gy (30 fractions)	60	Precise x-ray radiation beam (with imaging treatment planning).	
IMRT	2 Gy(30 fractions)	60	Use of a linear accelerator (LINAC) with x-rays, protons or other sources.	
VMAT	2 Gy (30 fractions)	60	Use of photons (x-rays) with high conformal dose distributions (improved target volume).	
WBRT	3–4 Gy (10 fractions)	30	X-ray radiation to the whole brain.	Promote anti-tumor response *via* IFN-β secretion which primes CD8^+^ cells, leading to tumor degradation however decreasing macrophages population.
SRT	5 Gy (6 fractions)	30	External high dose radiation using multiple, non-coplanar photon radiation beams.	
IGRT	6 Gy (10 fractions)	60	Radiation from x-rays, protons or other sources, using a LINAC or a cyclotron. Use of CT, MRI, ultrasound or x-ray to scan the tumor.	
FLASH-RT	10Gy (single pulse)	30	Single ultra-high-dose RT with 106 Gy/s with a low energy electron (LEE) prototype LINAC eRT6/Oriatron	Enhance the immune system by presenting more antigens. Increase levels of TREX1, inducing a reduction in the immune response. Decrease of proliferative capacity *via* increased levels of CXCL1 and CXCL5.
Internal selective RT	Depending on the radioisotope	60	Radioactive liquid treatment using a radioisotope or radionuclide. Bachytherapy technique.	Increase of IFN-β which activates T cells *via* MHCII.

Abbreviations: CD: cluster of differentiation; cGAS-STING: cyclic GMP-AMP synthase-stimulator of interferon genes; CRT: conformal radiation therapy; CT: computed tomography; CXCL: C-X-C motif ligand; IFN-β: interferon-β; IGRT: image-guided radiotherapy; IL: interleukin; IMRT: intensity modulated radiotherapy; MCP-1: monocyte chemoattractant protein 1; MHCII: major histocompatibility complex; MIP-1: macrophage inflammatory protein 1; 2MRI: magnetic resonance imaging; RT: radiotherapy; SRT: stereotactic radiotherapy; TREX1: three prime repair exonuclease 1; VMAT: volumetric modulated arc therapy; WBRT: whole brain radiotherapy.

**Table 2:** Ongoing clinical trials for immunotherapy of GBM associated with radiation therapy.

Clinical trial	Study population	Target	Intervention
NCT04047706 - Active NOT recruiting - (Phase 1)	Newly diagnosed GBM	IDO1	BMS-986205 – Nivolumab RTTMZ
NCT03532295 - Active NOT recruiting - (Phase 2)	Recurrent Gliomas		Epacadostat RT (35 Gy by 3.5 Gy fraction) Bevacizumab
NCT03661723 - Active NOT recruiting - (Phase 2)	Recurrent GBM	PD-1	Pembrolizumab – RT (35 Gy by 3.5 Gy fraction) Bevacizumab
NCT04977375 – Recruiting - (Phase 1/2)	Recurrent GBM		Pembrolizumab tereotactic Radiation (24 Gy in 8 Gy fraction) Surgical Resection
NCT03426891 – Completed - (Phase 1)	Newly diagnosed GBM		Pembrolizumab – Vorinostat RT (60 Gy in 2 Gy fraction) TMZ
NCT02617589 – Completed - (Phase 3)	Newly diagnosed GBM		Nivolumab RT TMZ
NCT02866747 - Active NOT recruiting - (Phase 1/2)	Recurrent GBM	PD-L1	Hypofractionated stereotactic radiation therapy (24 Gy by 8 Gy fraction) Durvalumab
NCT03174197 - Active NOT recruiting - (Phase 1/2)	Newly diagnosed GBM		Atezolizumab RT (60 Gy in 2Gy fraction) TMZ
NCT02968940 – Completed - (Phase 2)	GBM		Avelumab HFRT (30 Gy by 6 Gy fraction)
NCT02336165 – Completed - (Phase 2)	GBM		Durvalumab RT (60 Gy in 2 Gy fraction) Bevacizumab
NCT04922723 – Recruiting - (Phase 1/2)	GBM	CD-38	Daratumumab RT (60 Gy in 2 Gy fraction) TMZ
NCT04485949 – Recruiting - (Phase 2)	Newly diagnosed GBM	IGF-1 receptor	IGV-001 RT (60 Gy in 2 Gy fraction)
NCT02799238 – Completed - (Phase 2)	GBM	Tumor cells	ALECSAT RT (60 Gy in 2 Gy fraction) TMZ

Abbreviations: ALECSAT: Autologous Lymphoid Effector Cells Specific Against Tumor; CD: cluster of differentiation; GBM: glioblastoma; HFRT: hypofractionated radiotherapy; IGF-1: insulin-like growth factor; IDO1: indoleamine-pyrrole 2,3-dioxygenase; PD-1/PD-L1: programmed death ligand; RT: radiotherapy; TMZ: temozolomide.

## 6 Conclusion and perspectives

This review describes the impact of different irradiation regimes on the cellular microenvironment of GBM, in particular on GAMs and T cells. Fractionation between 1 and 2 Gy promotes the migration and maturation of immune cells (mainly macrophages), thereby enhancing the antitumor response. Between 3 and 6 Gy and 10 Gy, T-cell recruitment and activation are promoted by MHC class II and IFN-β secretion. Combined with immunotherapy, RT using adapted fractionation could lead to increased survival in GBM patients.

Given that RT appears to increase M2 infiltration in GBM [59] and that macrophages account for up to 30 % of infiltrating immune cells [90,91], several clinical studies are looking for new targets (other than blocking immune checkpoints such as PD-1) to inhibit maturation into an M2 phenotype. Immune (checkpoint) inhibitors such as anti-PD-1 are currently inconclusive in clinical studies. One of the main hypotheses to explain the lack of clinical benefit in GBM, is that research to date may have focused on the wrong target [92]. Indeed, drugs such as nivolumab, pembrolizumab and ipilimumab all reduce immune suppression mediated by Tregs by blocking either PD-1 or CTLA-4 [93,94]. Although Tregs play a crucial role in promoting CTL immunosuppression in many solid tumors, recent data suggest that this role is primarily played by GAM in GBM [95]. But that does not mean that CTLA-4 blockades isn't an interesting target for treating GBM. Indeed, CTLA-4 blockade was shown to stimulate microglia/macrophages phagocytosis through a cell partnership with Th1 cells which leads to anti-tumor function in GBM [96].

M2 macrophages are also present at higher percentages in GBM microenvironment compared to other tumor types, suggesting that they play a key role in immune suppression and may influence resistance to RT [15]. It is known that CSF-1 inhibition reduces M2 macrophage infiltration normally induced by conventional RT [97]. It is precisely for this reason that clinical trials are underway to inhibit CSF-1. Binding of CSF-1 to CSF-R1 triggers autophosphorylation on several tyrosine residues, which can activate multiple intracellular pathways, including phosphatidyl inositol 3-kinase (PI3K). Inhibition of CSF-R1 did not alter total macrophage numbers, but reduced M2 polarization; BLZ-945, a CSF-R1 inhibitor, reduces M2 infiltration in GBM and potentiates RT. This increased efficacy appears to be linked to the attenuation of M2-polarized infiltration, occurring in response to RT. Improved survival has been attributed to a decrease in microvessels density in tumor tissue due to a decrease in M2 population, widely characterized as pro-angiogenic. Their recruitment may also suppress cytotoxic activity of T lymphocytes [98,99]. To assess CSF-1 protein expression levels in clinics, and thus predict patient response to immunotherapy, a new approach called "immuno-PET" is currently being developed. Using an affibody against PD-L1, a small affinity ligand designed to mimic binding properties of antibodies coupled to a radiolabeled isotope, authors assessed PD-L1 expression levels by PET and monitored patient responses to immune checkpoint inhibitors [100].

In many solid tumors, Tregs play a crucial role in promoting CTL immunosuppression and moreover, their recruitment to the tumor microenvironment is significantly increased post-RT, resulting in inhibition of irradiation-induced anti-tumor immunity [101]. In addition, Tregs also appear to be radioresistant compared to other immune cells [102]. As they play a minor role in GBM immune suppression, clinical trials with immune checkpoint inhibitors specifically targeting Treg-mediated immune suppression mechanisms have been unsuccessful [92].

In a study entitled "Macrophage Exclusion after Radiation Therapy", authors evaluated a CXCR4 (CXCL12 receptor) inhibitor in a phase I/II clinical trial, involving 29 GBM patients. This chemokine, also known as SDF-1, promotes infiltration of inflammatory cells such as macrophages. By inhibiting CXCR4 (the CXCL12/CXCR4 axis is also involved in tumor progression, angiogenesis, metastasis, and survival), the authors succeeded in reducing macrophages infiltration and increasing overall survival to 21.3 months [103].

Valuable real-time assessment of cytokine and chemokine secretion pre- and post-RT could be necessary to comprehend time-dependent immunomodulatory effects of treatment on tumor microenvironment. An effective method available for this purpose is stereotactic microdialysis. This method was designed to monitor the interstitial tissue microenvironment through soluble factors samples using a semipermeable membrane at the tip of a microdialysis probe. It has previously been performed in GBM patients to detect metabolites both before and after RT [104,105]. An immediate inflammatory response was demonstrated in GBM after conventional RT(2 Gy fraction up to 60 Gy) [55]. Authors analyzed cytokines, glucose metabolites, glutamate and glycerol, suggesting that irradiation could rapidly enhance inflammation in GBM tumor tissue. Real-time monitoring of cytokines such as IFN-γ, CXCL1, MCP-1, IL-1β, TNF-α could be valuable for assessing the effects of the irradiation scheme on the GBM immune microenvironment, leading to a better understanding of the disease and potential therapeutic targets.

## CRediT authorship contribution statement

**Lucie Lerouge:** Writing – review & editing, Writing – original draft, Methodology, Conceptualization. **Aurélie Ruch:** Validation, Methodology, Investigation. **Julien Pierson:** Methodology, Investigation. **Noémie Thomas:** Writing – review & editing, Validation, Conceptualization. **Muriel Barberi-Heyob:** Writing – review & editing, Writing – original draft, Validation, Funding acquisition, Conceptualization.

## Declaration of competing interest

The authors have declared that no competing interest exists.

## Abbreviations

3D-CRT: three-dimensional conformal radiation therapy
APC: antigen-presenting cell
BBB: blood-brain barrier
BLZ-945: sotuletinib
BMDM: bone-marrow derived macrophages
CCL: CC motif chemokine ligand
CD: cluster differentiation
CNS: central nervous system
CRT: ER chaperone calreticulin
CSF-1: colony stimulating factor 1
CT: computed tomography
CTL: cytotoxic-T lymphocyte
CXCL: CXC motif chemokine ligand
DAMP: damage-associated molecular pattern
DNA: deoxyribose nucleic acid
EGF: epithelial growth factor
FSRT: fractionated stereotactic radiotherapy
GAL9: galectin-9
GAM: glioblastoma associated macrophages
GBM: glioblastoma
GM-CSF: granulocyte-macrophage colony stimulating factor
GSC: glioma stem cells
HFRT: hypofractionated radiotherapy
HIF-1α: hypoxia inducible factor 1α
HMGBI: high–mobility group box 1
IDH: isocitrate dehydrogenase
IDO1: Indoleamine-pyrrole 2,3-dioxygenase
IFN: interferon
IGF-1: insulin-like growth factor 1
IGRT: image-guided radiotherapy
IL: interleukin
IMRT: intensity-modulated radiotherapy
LPS: lipopolysaccharides
LT: lymphocyte T
MCP-1: monocyte chemoattractant protein 1
M-CSF: macrophage colony-stimulating factor
MDSC: myeloid-derived suppressor cell
MERT: macrophage exclusion after radiation therapy
MHC: major histocompatibility complex
MIC-1: macrophage inhibitory cytokine
MIP-1α/β: macrophage inflammatory protein
MMP: matrix metalloproteinase
MRI: magnetic resonance imaging
MRP-14: migration inhibitory factor-related protein 14
NABTT: new approaches to brain tumor therapy
PD-L1/PD-1: programmed death ligand
PET: positron tomography scan
ROS: reactive oxygen species
RT: radiotherapy
SCID: severe combined immunodeficient mice
SDF-1/CXCL12: stromal cell-derived factor 1
SRT: stereotactic radiotherapy
TCR: T cell receptor
TGF: tumor growth factor
TIGIT: T-Cell immunoreceptor with Ig and ITIM domain
TIM3: T-cell immunoglobulin and mucin-domain containing-3
TMZ: temozolomide
TNF: tumor necrosis factor
VEGF: vascular endothelial growth factor
VMAT: volumetric-modulated arc therapy
WBRT: whole brain radiotherapy
WHO: world health organization
